# Exploring knowledge and attitudes toward electronic informed consent among clinical trial participants in China: a cross-sectional study

**DOI:** 10.1186/s12910-025-01222-4

**Published:** 2025-05-26

**Authors:** Ying Wu, Xing Liu, Xiaoying Ge, Xin Tan, Weiwei Yu, Xiaomin Wang

**Affiliations:** 1 Center for Clinical Pharmacology, The Third Xiangya Hospital, Central South University, Changsha, 410013 Hunan People’s Republic of China; 2 School of Humanities, Central South University, Changsha, 410012 Hunan People’s Republic of China; 3 Xiangya Hospital of Central South University, Changsha, 410008 Hunan People’s Republic of China

**Keywords:** Research participant, Clinical research, Knowledge, Attitude

## Abstract

**Background:**

With the extensive integration of digital technology into clinical research, intelligence, virtualization, and decentralization have gradually transformed into emerging clinical research modes, the electronization of informed consent has become indispensable to the development of clinical trial informatization, and the inclination to use electronic informed consent (eIC) has grown. The knowledge and perceptions of research participants, as objects of informed consent acquisition, regarding eIC are crucial. However, few studies have empirically explored such issues.

**Methods:**

This cross-sectional study was conducted at three general hospitals in south-central China from July to September 2022. An online survey questionnaire was adapted and administered via WeChat to investigate the issues of interest.

**Results:**

A total of 388 valid questionnaires were included in the analysis. The results showed that the overall response rate for the knowledge section of the questionnaire exceeded 70%. Of the respondents, 53.1% had heard of the term “electronic informed consent,” but only 43.2% had used eIC. The majority of respondents (68%) expressed a preference for using eIC and demonstrated a positive attitude toward it. However, some participants expressed concerns regarding the security and confidentiality (64.4%), operational complexity (52.3%), and effectiveness of online interaction (59.3%) in eIC. Statistically significant relationships were observed between participants’ attitude scores and their age, gender, type of participation, and frequency of involvement in clinical research. Additionally, a positive and statistically significant correlation was found between participants’ knowledge scores and their attitude scores.

**Conclusion:**

The results of this study indicate that most participants have a good understanding of eIC-related knowledge and hold a positive attitude toward its implementation. However, they also express concerns about data protection and privacy security in eIC. These findings provide a foundation for developing targeted strategies to enhance the adoption and acceptance of eIC among diverse populations.

## Background

The Belmont Report outlines ethical principles and guidelines for research involving human participants. It identifies three key elements that must be ensured for genuine informed consent: information, comprehension, and voluntariness [1]. While informed consent process has become increasingly regulated and standardized in recent years, persistent structural challenges remain unresolved and demand urgent attention [2]. For example, the increasing length, complexity, and incorporation of legal language into consent forms render them less likely to be read or understood. Studies have shown that even after signing consent forms, participants frequently demonstrate limited understanding of study information [2]. In addition, the traditional face-to-face acquisition of informed consent suffers from certain regional limitations that prevent many eligible individuals from participating [3].

The proliferation of digital innovations is fundamentally transforming every facet of healthcare delivery [4]. Electronic health records have become ubiquitous in clinical practice, while patient-provider interactions are increasingly being conducted through digital channels [5]. This technological shift creates unprecedented opportunities to revolutionize informed consent process [6]. Electronic informed consent (eIC) refers to the use of electronic systems and processes that employ various electronic media, including text, images, audio and video, passive and interactive websites, biometric devices, and card readers, among others, to convey research-related information and to obtain and record informed consent [7]. In summary, eIC comprises two components: e-informing and e-consenting. E-informing involves delivering study-related information to participants through diverse digital formats, which provide greater flexibility compared to traditional paper-based methods. E-consenting specifically denotes the process of obtaining legally valid consent via electronically executed signatures.

Currently, eIC has been preliminarily implemented in clinical settings such as cancer treatment centers [6]and trauma and orthopedic departments [8]. Its core value lies in reconstructing traditional informed consent processes through digital tools. On the one hand, in decentralized clinical trials (DCTs), eConsent technology enables remote online signing, significantly reducing or even eliminating subjects’ physical reliance on trial sites, thereby serving as critical infrastructure for DCT operations [9]. On the other hand, electronic platforms provide multidimensional opportunities to improve the informed consent procedures. For example, by integrating functional modules such as visual information presentation (graphics/video), adaptive content delivery, interactive decision support, and on-demand information expansion, these platforms systematically enhance subjects’ comprehension of study protocols and their engagement satisfaction [10].

Given the significant advantages of eIC in enhancing the efficiency of clinical trials and optimizing ethical review processes, multiple countries around the world have successively introduced supportive policies to actively promote the standardized application of eIC in clinical trials. In 2016, the United States Food and Drug Administration (FDA) issued the guidance titled “Use of Electronic Informed Consent in Clinical Investigations - Questions and Answers,” which provides recommendations on the use of electronic systems and processes [7]. Similarly, in 2018, the United Kingdom’s Medicines and Healthcare products Regulatory Agency (MHRA) and Health Research Authority (HRA) released a joint statement outlining the legal and ethical requirements for obtaining and recording consent electronically [11]. In 2020, the National Medical Products Administration (NMPA) of China issued the “Guidelines for the Management of Drug Clinical Trials During the COVID-19 Pandemic”, formally incorporating electronic informed consent forms into the clinical trial management system. This provided institutional safeguards for protecting the rights and interests of participants in remote healthcare scenarios [12].

Nowadays, research on the development of eIC in China is relatively limited, particularly in terms of clinical research participants’ perceptions and attitudes toward eIC, which have not yet been fully explored. Our previous study compared the attitudes toward eIC between participants with prior clinical trial experience and those who agreed to participate in clinical trial recruitment [13]. The results revealed that participants with clinical trial experience had significantly more positive attitudes toward eIC, suggesting a potential link between prior participation experience and eIC acceptance. Based on these findings, this study further focuses on clinical trial participants (including both patients and healthy volunteers), aiming to thoroughly validate the relationship between “participation experience” and “eIC acceptance.”

## Methods

### Study design and participants

This cross-sectional study was conducted at three general hospitals in south-central China, specifically in Changsha, the capital city of Hunan Province, from July to September 2022. Participants who met the following inclusion criteria were recruited: (1) at least 18 years of age, (2) experience taking part in one or more clinical studies, and (3) willing to participate in the present research, as signified by their provision of informed consent.

We first distributed a questionnaire link via WeChat to researchers working at the three general hospitals, after which these researchers distributed the questionnaire link to the participants for completion.

### Questionnaire

The questionnaire, called the Electronic Informed Consent Attitude Scale (eIC Attitude Scale), was adapted from a survey instrument that we previously developed for clinical trial research participants [13]. Its readability and comprehensibility were improved to enhance the accuracy of results. The adapted questionnaire consists of five sections: The first contains the informed consent form, and the second section revolves around demographic information. The third section focuses on basic information about eIC use and comprises single- and multiple-choice questions. The fourth section is a knowledge survey that consists of eight questions about the correctness of eIC statements, with a participant presented with the options of “yes,” “no,” and “don’t know.” Correct answers were scored 1 point, and the rest were scored 0. The fifth section is a self-report attitude survey comprising 17 statements intended to measure participants’ attitudes toward eIC. The statements are rated on a five-point Likert scale ranging from 1 (*strongly disagree*) to 5 (*strongly agree*points). For descriptive purposes, we summed the percentages of *strongly agree* and *agree* responses, as well as the percentages of *strongly disagree* and *disagree* responses. In calculating the scores for the eIC Attitude Scale, five statements were negatively worded and reverse-scored (e.g., a score of 5 was converted into a score of 1, and vice versa).

### Data analysis

Data was analyzed using SPSS 26.0. The reliability of the eIC Attitude Scale was tested by calculating the Cronbach’s alpha coefficient of the scale, which was 0.820, indicating good internal consistency(0.8–0.9). Both descriptive and inferential statistics were utilised. Categorical variables were described using frequencies and percentages, and the median with interquartile range (IQR) were used for continuous variables. The associations between attitude scores and respondents’ characteristics were assessed using the nonparametric Mann-Whitney U test and Kruskal–Wallis test. The correlation between knowledge scores and attitude scores was calculated using the Spearman’s correlation. A significance level of *P* < 0.05 was considered statistically significant.

## Results

This study collected a total of 529 questionnaires, with 388 valid responses, yielding an effective response rate of 73.4%. Demographic characteristics of the sample revealed a higher proportion of female participants compared to a relatively lower proportion of males. In terms of participant types, the patient group constituted the majority, while healthy volunteers accounted for a smaller proportion. The vast majority of participants (86.9%) preferred mobile devices as their primary choice for accessing electronic informed consent. Approximately 53.1% of participants reported having heard of electronic informed consent, among whom less than half (43.2%) had actually used it. Regarding economic status, more than half of the participants reported a monthly income exceeding 6,000 RMB, a level significantly higher than the per capita disposable income of residents in Changsha City [14]. (Table 1)

**Table 1:** Participant characteristics (*n* = 388)

Characteristics	*N* (%)
**Age**	
18–30 years	146(37.6)
31–40 years old	136(35.1)
41 years old and above	106(27.3)
**Sex**	
Male	75(19.3)
Female	313(80.7)
**Academic qualifications**	
High school and below	71(18.3)
Junior college/college	96(24.7)
Bachelor’s degree	162(41.8)
Master’s degree and higher	59(15.2)
**Place of residence**	
City (including county/town)	355(91.5)
Rural	33(8.5)
**Personal monthly income level**	
Less than 4000 RMB	80(20.6)
4000–5999 RMB	108(27.8)
6000–7999 RMB	83(21.4)
Above 8000 RMB	117(30.2)
**Type of participation in clinical studies**	
Healthy volunteers	145(37.4)
Patients	243(62.6)
**Frequency of participation**	
Once	297(76.5)
≥2 times	91(23.5)
**Had heard of electronic informed consent**	
Yes, I have.	206(53.1)
Never heard of it	182(46.9)
**Had used electronic informed consent (*n*** **= 206)**	
Yes	89(43.2)
No	117(56.8)
**Preferred device when giving electronic informed consent**	
PC	51(13.1)
Mobile device (e.g., cell phone or tablet)	337(86.9)

**Fig. 1:**
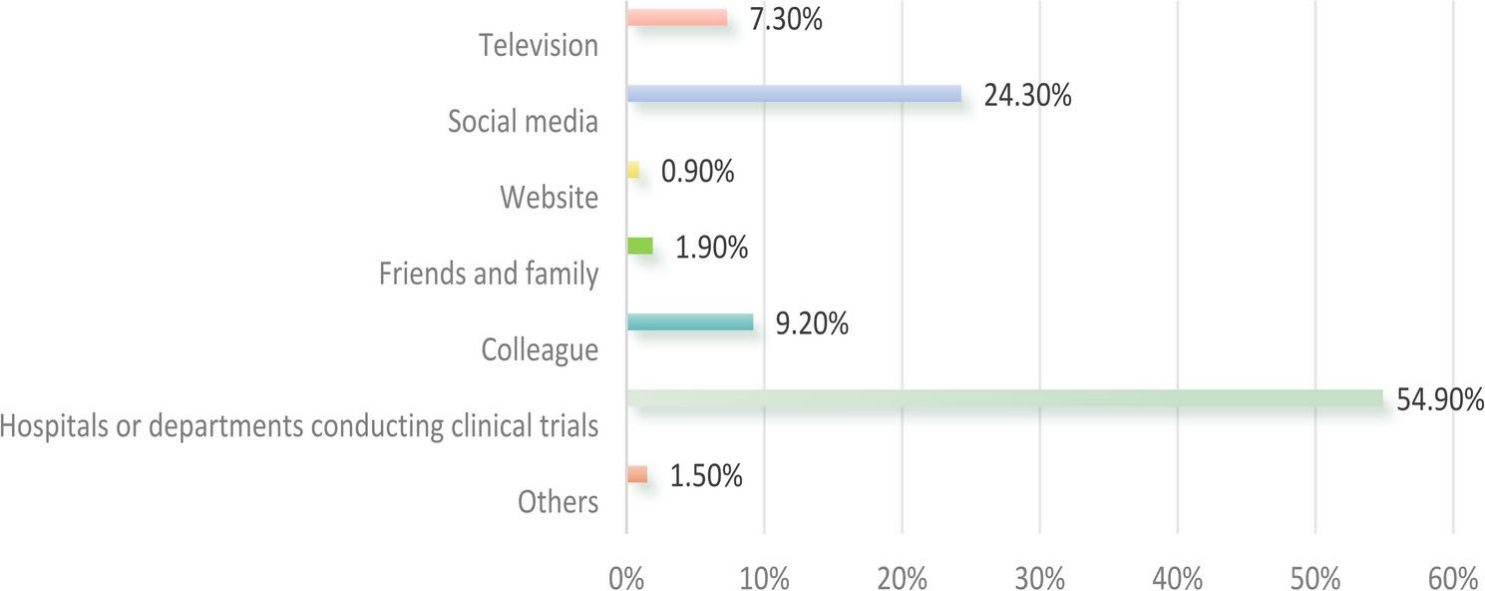
Primary channels through which participants heard about electronic informed consent (*n* = 206)

**Fig. 2:**
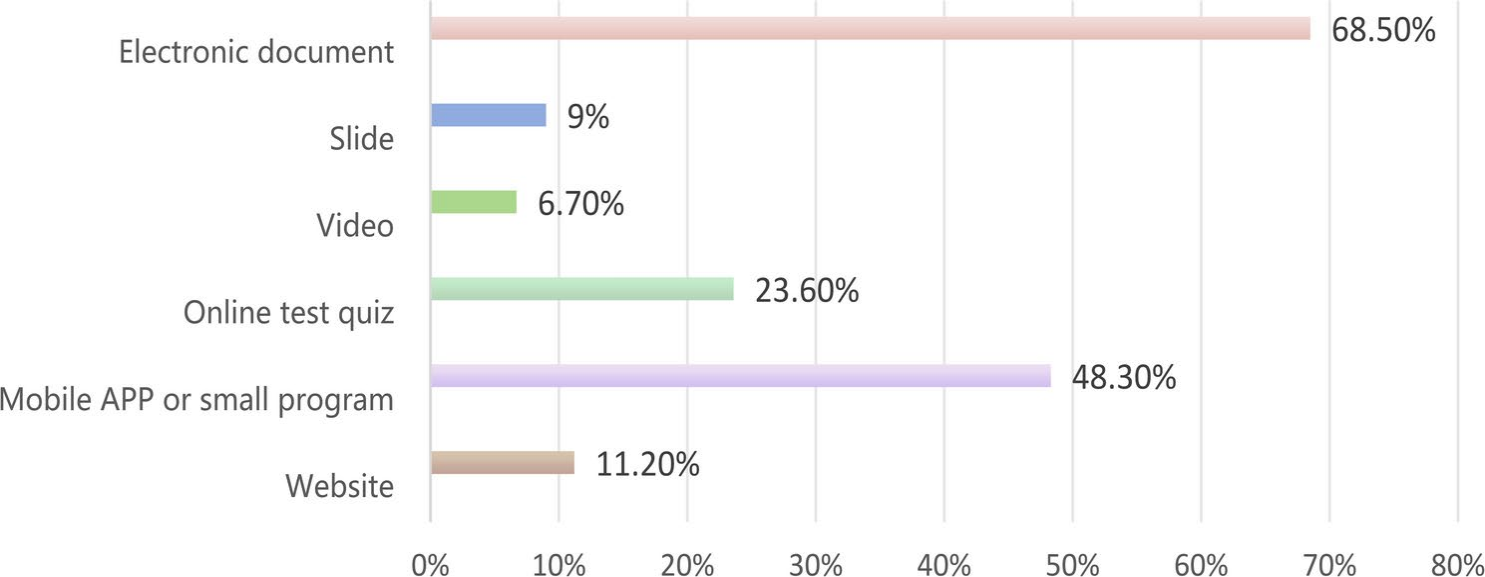
Forms of using electronic informed consent (*n* = 89)

Regarding the primary channels through which the participants heard about eIC, ahospitals or clinical trial departments accounted for the largest proportion at 54.9% (Fig. 1). Among the participants who had used eIC, the most prevalent format was electronic documents, accounting for 68.5%, followed by mobile applications or small programs at 48.3% (Fig. 2).

When responding to questions related to eIC knowledge, participants demonstrated an overall accuracy rate ranging from 71 to 81%. Specifically, over 75% of the participants correctly answered a series of questions related to eIC (Q1-4, 6–8). These questions encompassed fundamental concepts of eIC, the diversity of electronic media used, methods of patient–physician communication, the reliability and application of electronic signatures, and ethical review requirements. However, only 71.4% of the participants were aware of the flexibility in usage location of electronic medical records, indicating no spatial constraints (Q 5), reflecting a relatively lower level of awareness in this aspect (Table 2).

**Table 2:** Knowledge of participants toward electronic informed consent

Question	Yes (%)
Q1: eIC utilizes electronic systems and software to digitally deliver clinical research information and record the informed consent process.	311(80.2)
Q2: eIC employs multimedia interfaces (e.g., videos, quizzes) and interactive modules to enhance subjects’ comprehension of clinical study information.	311(80.2)
Q3: eIC forms contain all elements identical to paper-based informed consent form.	316(81.4)
Q4: eIC platforms may integrate multimedia formats (e.g., documents, images, audio/video files) and interactive tools (e.g., web-based quizzes, digital signature pads), with optional biometric authentication.	307(79.1)
Q5: The eIC process may occur in two modalities: on-site (conducted in-person at a clinical facility with a study physician) or remote (completed through a secured digital platform without physical presence of study staff).	277(71.4)
Q6: Communication during eIC must align with the selected modality: on-site requires face-to-face discussions with study personnel, whereas remote exclusively utilizes electronic devices (e.g., video calls, online chats).	299(77.1)
Q7: An electronic signature must be voluntarily provided by the subject or their legally authorized representative (e.g., court-appointed guardian).	296(76.3)
Q8: The use of eIC requires a review and approval by an ethics committee.	293(75.5)

The majority of participants demonstrated positive attitudes toward eIC, with a significant proportion expressing willingness to adopt the technology and viewing it as more convenient and efficient compared to traditional paper-based consent. Over half believed that eIC could fully replace paper-based consent, and a large majority showed openness to advanced technologies like facial and fingerprint recognition for e-signatures. However, despite these positive views, a notable portion of participants raised concerns regarding the security and confidentiality of eIC. Many were apprehensive about the complexity of its operation and doubted the effectiveness of online interactions compared to face-to-face engagement. Additionally, a substantial majority emphasized the need for clearer explanations of privacy protection and data confidentiality measures within eIC (Table 3).

**Table 3:** Attitudes of participants toward electronic informed consent

Attitude statements	Strongly Agree (%)	Agree (%)	Not sure (%)	Disagree (%)	Strongly disagree (%)	Median (IQR)
1. I prefer eIC over paper-based informed consent.	106 (27.3)	158 (40.7)	89 (22.9)	13 (3.4)	22 (5.7)	3.41 (3.12–3.82)
2. I think eIC is more convenient than paper-based informed consent.	119 (30.7)	172 (44.3)	63 (16.2)	13 (3.4)	21 (5.4)	
3. The use of eIC is more efficient (e.g., time and resource savings) than paper-based informed consent.	120 (30.9)	174 (44.8)	59 (15.2)	13 (3.4)	22 (5.7)	
4. I think eIC can completely replace paper-based informed consent.	98 (25.3)	131 (33.8)	84 (21.6)	48 (12.4)	27 (7.0)	
5. I can accept the use of technologies such as face recognition and fingerprint recognition for eIC.	107 (27.6)	173 (44.6)	62 (16.0)	21 (5.4)	25 (6.4)	
6. I am worried that the operation method of eIC (e.g., interface design, user-friendliness) is too complicated to enable me to adapt to its use.	61 (15.7)	142 (36.6)	73 (18.8)	64 (16.5)	48 (12.4)	
7. I have concerns about the security and confidentiality (e.g., privacy protection, data breach risks) of eIC compared with paper-based informed consent.	77 (19.8)	173 (44.6)	75 (19.3)	29 (7.5)	34 (8.8)	
8. I have concerns about the legal validity and protection of rights and interests under eIC.	72 (18.6)	161 (41.5)	80 (20.6)	34 (8.8)	41 (10.6)	
9. Even at a research site, I prefer to use electronic devices (e.g., computer, cell phone) for eIC than paper-based informed consent.	90 (23.2)	159 (41.0)	86 (22.2)	31 (8.0)	22 (5.7)	
10. I am more willing to participate in eIC remotely than eIC on-site (i.e., not at a research site).	96 (24.7)	158 (40.7)	81 (20.9)	33 (8.5)	20 (5.2)	
11. I am concerned that research subjects will not understand a study when using eIC (e.g., purpose of the study, research methods).	57 (14.7)	163 (42.0)	77 (19.8)	50 (12.9)	41 (10.6)	
12. I think the eIC process is more helpful in setting up interactions with research subjects (e.g., online Q&A), aiding them in comprehending research information.	101 (26.0)	191 (49.2)	66 (17.0)	12 (3.1)	18 (4.6)	
13. I am concerned that online interaction with research subjects using eIC is not as effective as face-to-face interaction.	62 (16.0)	168 (43.3)	87 (22.4)	38 (9.8)	33 (8.5)	
14. I think that a more detailed description of privacy protection and data confidentiality measures should be added to eIC.	118 (30.4)	191 (49.2)	56 (14.4)	5 (1.3)	18 (4.6)	
15. The use of eIC eases subject decision-making regarding whether to participate in a clinical trial.	84 (21.6)	145 (37.4)	114 (29.4)	25 (6.4)	20 (5.2)	
16. I believe that the use of eIC provides better guidance to research subjects in signing informed consent forms and avoiding errors and omissions.	92 (23.7)	165 (42.5)	93 (24.0)	21 (5.4)	17 (4.4)	
17. I think the regulations related to eIC for clinical in China trials should be further improved.	133 (34.3)	192 (49.5)	43 (11.1)	2 (0.5)	18 (4.6)	

IQR = Interquartile range

Younger participants (aged 18–30) exhibited significantly higher attitude scores compared to older participants (aged 31 and above) (*p* < 0.05). Male participants scored higher in attitude than female participants (*p* < 0.05). Participants who joined the clinical study as healthy volunteers had higher scores than those who participated as patients (*p* < 0.05). Additionally, participants who had engaged in multiple clinical studies showed higher attitude scores compared to those who had participated only once (*p* < 0.05) (Table 4).

**Table 4:** The effect of different characteristics of participants on attitude scores on electronic informed consent

Variables	*N* (%)	Median (IQR)	^a^*p*-value
**Age**			
18–30 years	146 (37.6)	3.47 (3.24–3.82)	0.006^*^
31–40 years old	136 (35.1)	3.41 (3.00–3.69)	
41 years old and above	106 (27.3)	3.41 (3.12–3.60)	
18–30 years old vs. 31–40 years old			0.011^*^
18–30 vs. 41+			0.040^*^
31–40 vs. 41+			1
**Sex**			
Male	75(19.3)	3.65 (3.35–3.94)	0.000^*^
Female	313 (80.7)	3.41 (3.12–3.65)	
**Academic qualifications**			
High school and below	71 (18.3)	3.35 (3.06–3.71)	0.401
Junior college/college	96 (24.7)	3.47 (3.18–3.82)	
Bachelor’s degree	162 (41.8)	3.41 (3.18–3.65)	
Master and higher	59 (15.2)	3.41 (3.06–3.82)	
**Place of residence**			
City (including county/town)	355 (91.5)	3.41 (3.12–3.82)	0.706
Rural	33(8.5)	3.35 (3.03–3.82)	
**Personal monthly income level**			
Less than 4000 RMB	80 (20.6)	3.41 (3.06–3.65)	0.633
4000–5999 Yuan	108 (27.8)	3.41 (3.12–3.82)	
6000–7999 Yuan	83 (21.4)	3.41 (3.12–3.71)	
More than 8000 RMB	117 (30.2)	3.41 (3.12–3.82)	
**Types of participation in clinical studies**			
Healthy volunteers	145(37.4)	3.59 (3.24–3.97)	0.000^*^
Patients	243(62.6)	3.41 (3.06–3.53)	
**Frequency of participation**			
1	297 (76.5)	3.41(3.12–3.65)	0.000^*^
≥ 2	91 (23.5)	3.65 (3.24–4.06)	

^a^Mann–Whitney U and Kruskal–Wallis tests were carried out

^*^Statistically significant

IQR = Interquartile range

We assessed the correlation between knowledge scores and attitude scores through Spearman’s correlation analysis. It was found that the two scores were positively correlated and statistically significant (*r* = 0.274, *p* < 0.05).

## Discussion

The results indicate that participants’ knowledge of eIC is at a good level. Most participants hold a positive attitude towards eIC, while expressing concerns about its security and confidentiality, the complexity of operation, and the effectiveness of online interaction. There is a statistically significant relationship between participants’ different characteristics, such as age, gender, type of participation, and frequency of participation in clinical research, and their attitude scores. Additionally, there is a positive and statistically significant relationship between participants’ knowledge scores and attitude scores.

Nearly half of the participants (patients who have participated in clinical trials and healthy volunteers) have not heard of eIC and report a low usage rate, a result consistent with our previous study (participants with clinical trial experience and those who agreed to participate in clinical trial recruitment) [13]. This consistency suggests that the limited awareness of eIC may stem from its low application rate and insufficient public promotion, rather than specific population characteristics. Furthermore, consistent with the findings of this study, in the research by Corneli et al. (2024), participants held a positive attitude towards eIC, finding digital elements to be humane and making them feel more informed, engaged, comfortable, and prepared when participating in clinical research [15]. Govil et al. (2023) found that patients’ satisfaction with eIC was higher than that of employees, believing that eIC facilitates fully informed consent [16]. Guarino et al. (2022) also showed that compared to traditional paper consent forms, electronic consent forms improved patients’ usability, satisfaction, knowledge, and trust scores [17].

The study findings reveal statistically significant differences in attitude scores based on four key demographic variables: age, frequency of participation and type of participation in clinical studies, gender. In comparison with our previous research, this study incorporated additional stratification variables (such as age groups and frequency of clinical trial participation). The results demonstrate that younger participants and those involved in multiple clinical trials exhibit higher acceptance levels of eIC compared to older individuals and those participating in clinical trials for the first time. These findings highlight the necessity for targeted interventions tailored to specific demographic groups. For instance, developing more user-friendly eIC interfaces for elderly populations or first-time trial participants could be a strategic approach. Consistent with these findings, Arning et al. (2009) also identified age as a significant factor influencing acceptance ratings, noting discernible differences in the acceptance of electronic health technologies among various age groups [18]. The results revealed that healthy volunteers exhibited significantly higher attitude scores towards eIC compared to patients (*P* < 0.05). This difference may be attributed to the proactive engagement of healthy volunteers in clinical research, which likely enhances their confidence in understanding eIC. Individuals who regularly participate in trials have higher attitude scores (*P* < 0.05), and they generally have a more favorable view of clinical research, which may influence their positive attitudes toward eIC. Additionally, male participants demonstrated higher eIC attitude scores than their female counterparts (*P* < 0.05), suggesting potential gender-based differences in perceptions of electronic product usage. This finding aligns with previous research indicating that men tend to hold more positive attitudes toward computer-related or digital technologies compared to women [19, 20]. However, it is important to note that in our study, male participants accounted for only one-quarter of the total sample size. Due to this limited representation, the generalizability of these findings may be constrained.

In our study, some participants expressed concerns regarding the operational complexity, interactive effectiveness, as well as the security and confidentiality of the eIC system. This finding aligns with previous research. Spencer et al. (2016) noted that a significant proportion of patients were apprehensive about using touchscreen interfaces in eIC, particularly those with limited technical knowledge, and felt they might require assistance to navigate the system [21]. Similarly, Mazzochi et al. (2023) highlighted that the complexity of eIC processes or operations could potentially hinder the recruitment of clinical research participants [22]. Furthermore, Wood et al. (2011) emphasized the concerns of middle-aged participants regarding the privacy and security of online consent procedures [23]. Additionally, Simon et al. (2018), through interviews with underrepresented patient groups, found that these populations also harbored reservations about the accessibility, connectivity, privacy, and confidentiality aspects of eIC [24].

The development of eIC faces several challenges, particularly concerning security and confidentiality, which necessitate collaborative efforts from all relevant stakeholders, including Institutional Review Boards (IRBs), researchers, sponsors, research institutions, and policymakers. To establish a standardized framework for eIC usage and ethical review in China, it is recommended that a national regulatory body or the National Medical Ethics Association take the lead in forming an interdisciplinary expert group. This group should encompass expertise in ethics, law, information technology, and clinical research, and be tasked with developing technical standards, usage guidelines, and an ethical review framework for eIC. Furthermore, it is advisable to create adaptable eIC tools tailored to the preferences of research participants. For instance, developing videos and interactive Q&A modules designed for different demographic groups could enhance user engagement and comprehension. Additionally, leveraging existing infrastructure, such as hospital patient education platforms and clinical trial registration systems, to integrate eIC educational content could facilitate broader dissemination and adoption. This multi-faceted approach would not only address the technical and ethical challenges associated with eIC but also promote its effective implementation across diverse research settings.

This study has several limitations. First, the sample was primarily drawn from three general hospitals in Hunan Province, which may limit the representativeness of the findings and affect their generalizability. Second, the participants generally had a higher level of education, which means the perspectives of groups with lower educational attainment were not adequately represented. Third, the study focused exclusively on participants with clinical research experience, leaving out other relevant groups. To address these limitations, future research should aim to expand the sample size and include a more diverse population, particularly incorporating the perspectives of key stakeholders such as researchers, health administration departments, ethics committees, and eIC platform developers.

## Conclusion

In general, the findings of the study show that the majority of participants demonstrated high understanding of eIC’s related concepts and held positive attitudes toward its implementation. The participants’ age, gender, number of clinical trial participations, and types of participation in clinical studies are factors that may influence their attitudes toward eIC. A significant correlation was observed between participants’ knowledge of eIC and their acceptance levels, with higher knowledge associated with greater acceptance. This research further validates the potential association between ‘participation experience’ and ‘eIC acceptance,’ while identifying target groups for future interventions—such as middle-aged/older adults and individuals with no or limited clinical trial experience—through the inclusion of additional variables (e.g., age stratification, frequency of clinical trial participation).

## Data Availability

The datasets used and/or analyzed during the current study are available from the corresponding author on reasonable request.

## Abbreviations

eIC: Electronic informed consent
DCTs: Decentralized clinical trials
FDA: Food and drug administration
HRA: Health research authority
IQR: Interquartile range
IRBs: Institutional review boards
MHRA: Medicines and healthcare products regulatory agency
NMPA: National medical products administration
